# Supplementary material for: Psoriatic arthritis screening: A systematic literature review and experts’ recommendations
Source: PLoS One. 2021 Mar 15;16(3):e0248571. doi: 10.1371/journal.pone.0248571 (PMC7959352; doi:10.1371/journal.pone.0248571)
Supplement: S1 File — (DOCX) [file pone.0248571.s002.docx]

**S1 Table**. Medline search strategy.

| # | Search terms | Citations |
| --- | --- | --- |
| **1** | "Arthritis, Psoriatic"[MH] | 5.853 |
| **2** | “Psoriatic arthritis” [tw] | 9.356 |
| **3** | “Psoriasis, Arthritic” OR “Arthritic Psoriasis” OR “Psoriatic Arthritis” OR “Psoriasis Arthropathica” OR “Psoriatic Arthropathy” OR “Arthropathies, Psoriatic” OR “Arthropathy, Psoriatic” OR “Psoriatic Arthropathies” | 10.301 |
| **4** | #1 OR #2 OR #3 | 10.301 |
| **5** | “Toronto Psoriatic Arthritis Screen” OR “ToPAS” OR “Toronto Psoriatic Arthritis Screen II” OR “ToPAS II” OR “the Psoriatic Arthritis Screeningand Evaluation” OR “PASE” OR “the Psoriasis Epidemiology Screening Tool” OR “PEST” OR “the Early Arthritis for Psoriatic Patients” OR “EARP” OR “Simple Psoriatic Arthritis Screening” OR “SiPAS” OR “German Psoriasis Arthritis Diagnostic” OR “GEPARD” OR "PsA-Disk" | 40.303 |
| **6** | "Surveys and Questionnaires"[MH] | 998.071 |
| **7** | "Early Diagnosis"[MH] | 48.734 |
| **8** | "Referral and Consultation"[MH] | 73.011 |
| **9** | “Classification criteria for psoriatic arthritis” OR “CASPAR” OR “CONTEST” | 3.402 |
| **10** | #5 OR #6OR #7 OR #8 OR #9 | 1.138.593 |
| **11** | Randomized [All Fields] OR random allocation”[TIAB] NOT Medline[SB]) OR “random allocation”[MH] OR randomized [TW] OR controlled [All Fields] AND (“clinical trials as topic”[MH] OR trial [TW]) OR placebo OR blinded OR randomized controlled trial [Publication Type] | 1.252.313 |
| **12** | "Epidemiologic Studies"[MH] | 2.421.529 |
| **13** | "Systematic Reviews as Topic"[MH] | 2.960 |
| **14** | "Meta-Analysis as Topic"[MH] | 18.714 |
| **15** | "Sensitivity and Specificity"[MH] | 570.138 |
| **16** | #11 OR #12 OR #13 OR #14 OR #15 | 3.791.440 |
| **17** | #4 AND #10 AND #16 | 1.239 |
| **18** | (#4 AND #10 AND #16) Filters: Humans; English; Spanish | 1.162 |

**S2 Table**. Embase search strategy.

| # | Search terms | Citations |
| --- | --- | --- |
| **1** | 'psoriatic arthritis'/exp | 21.516 |
| **2** | 'arthritis psoriatica' OR 'arthritis, psoriatic' OR 'arthritis, psoriasis' or 'arthritis, psoriatic' or 'arthropathic psoriasis' or 'arthropathy, psoriatic' or 'polyarthritis, psoriatic' or 'psoriasis arthropathica' or 'psoriasis pustulosa arthropathica' or 'psoriasis, arthritis' or 'psoriatic arthropathy' or 'psoriatic polyarthritis' | 2.517 |
| **3** | #1 OR #2 | 22.329 |
| **4** | 'toronto psoriatic arthritis screen' OR 'topas' OR 'toronto psoriatic arthritis screen ii' OR 'topas ii' OR 'the psoriatic arthritis screeningand evaluation' OR 'pase' OR 'the psoriasis epidemiology screening tool' OR 'pest' OR 'the early arthritis for psoriatic patients' OR 'earp' OR 'simple psoriatic arthritis screening' OR 'sipas' OR 'german psoriasis arthritis diagnostic' OR 'gepard' OR 'psa-disk' | 45.136 |
| **5** | 'psoriatic arthritis screening and evaluation questionnaire'/exp | 15 |
| **6** | 'psoriatic arthritis screening and evaluation'/exp | 19 |
| **7** | 'early diagnosis'/exp | 103.035 |
| **8** | 'patient referral'/exp | 110.439 |
| **9** | 'classification criteria for psoriatic arthritis' OR 'caspar' OR 'contest' | 6.176 |
| **10** | #4 OR #5 OR #6 OR #7 OR #8 OR #9 | 262.604 |
| **11** | #3 AND #10 | 1.499 |

**S3 Table**. Cochrane Library search strategy.

| # | Search terms | Citations |
| --- | --- | --- |
| **1** | Mesh descriptor: "Arthritis, Psoriatic" explode all trees | 364 |
| **2** | Psoriatic arthritis | 2.089 |
| **3** | “Psoriasis, Arthritic” OR “Arthritic Psoriasis” OR “Psoriatic Arthritis” OR “Psoriasis Arthropathica” OR “Psoriatic Arthropathy” OR “Arthropathies, Psoriatic” OR “Arthropathy, Psoriatic” OR “Psoriatic Arthropathies” | 2.052 |
| **4** | #1 OR #2 OR #3 | 2.100 |
| **5** | “Toronto Psoriatic Arthritis Screen” OR “ToPAS” OR “Toronto Psoriatic Arthritis Screen II” OR “ToPAS II” OR “the Psoriatic Arthritis Screeningand Evaluation” OR “PASE” OR “the Psoriasis Epidemiology Screening Tool” OR “PEST” OR “the Early Arthritis for Psoriatic Patients” OR “EARP” OR “Simple Psoriatic Arthritis Screening” OR “SiPAS” OR “German Psoriasis Arthritis Diagnostic” OR “GEPARD” OR "PsA-Disk" | 5.702 |
| **6** | Mesh descriptor: "Mass Screening" explode all trees | 3.710 |
| **7** | Mesh descriptor: "Surveys and Questionnaires" explode all trees | 52.988 |
| **8** | Mesh descriptor: " Early Diagnosis" explode all trees | 1.623 |
| **9** | Mesh descriptor: " Referral and Consultation" explode all trees | 2.229 |
| **10** | “Classification criteria for psoriatic arthritis” OR “CASPAR” OR “CONTEST” | 373 |
| **11** | #1 or #2 or #3 | 2.100 |
| **12** | #4 or #5 or #6 or #7 or #8 or #9 or #10 | 108.444 |
| **13** | #11 and #12 in Cochrane Reviews, Trials, Clinical Answers and Special collections | 423 |

**S4 Table.** Excluded studies and reasons for exclusion.

| # | Study | Exclusion reason |
| --- | --- | --- |
| 1 | Bachelez_2011 [1] | French language |
| 2 | Barbour_2003 [2] | The screening tool is for inflammatory joint disease |
| 3 | Biondi_2018 [3] | Specific data on diagnostic performance are not provided |
| 4 | Brzezińska_2018 [4] | Polish language |
| 5 | Busquets-Perez_2015 [5] | Narrative review |
| 6 | Cantini_2012 [6] | Specific data on screening tools are not provided |
| 7 | Chandran_2008 [7] | Specific data on screening tools are not provided |
| 8 | Choe_2019 [8] | Specific data on diagnostic performance are not provided |
| 9 | Coates_2017 [9] | Opinion article |
| 10 | García-Gavín_2017 [10] | Specific data on diagnostic performance are not provided |
| 11 | Gladman_2008 [11] | Narrative review |
| 12 | Henes_2014 [12] | Specific data on diagnostic performance are not provided |
| 13 | Iragorri_2019 [13] | SLR. The included articles are already selected in our SLR |
| 14 | Jo_2019 [14] | Specific data on diagnostic performance are not provided |
| 15 | Khraishi_2012 [15] | Specific data on screening tools are not provided |
| 16 | Mease_2019 [16] | Specific data on screening tools are not provided |
| 17 | Merola_2013 [17] | Specific data on screening tools are not provided |
| 18 | Merola_2014 [18] | Opinion article |
| 19 | Olivieri_2008 [19] | Opinion article |
| 20 | Ravi_2019 [20] | Specific data on screening tools are not provided |
| 21 | Salaffi_2017 [21] | This is a pilot study of another that is included |
| 22 | Shin_2016 [22] | Specific data on screening tools are not provided |
| 23 | Spelman_2015 [23] | Specific data on diagnostic performance are not provided |
| 24 | Tay_2014 [24] | Specific data on screening tools are not provided |
| 25 | Taylor_2009 [25] | Recommendations document |
| 26 | Wernham_2015 [26] | Specific data on screening tools are not provided |
| 27 | Wittkowski_2011 [27] | Specific data on screening tools are not provided |

**Abbreviations**: SLR=systematic literature review.

**S5 Table.** QUADAS-2 results of the included studies.

| **Study**  Audureau_2018 [28] | **RISK OF BIAS** | | | | **APPLICABILITY CONCERNS** | | |
| --- | --- | --- | --- | --- | --- | --- | --- |
|  | **PATIENT SELECTION** | **INDEX TEST** | **REFERENCE STANDARD** | **FLOW AND TIMING** | **PATIENT SELECTION** | **INDEX TEST** | **REFERENCE STANDARD** |
| Chandran_2011 [29] | ☺ | ☺ | ☺ | ☺ | ☺ | ☺ | ☺ |
| Chimenti_2019 [30] | ☺ | ☺ | ☹ | ☺ | ☺ | ☺ | ☹ |
| Chiowchanwisawakit_2016 [31] | ? | ☺ | ☺ | ☺ | ? | ? | ☺ |
| Coates_2013 [32] | ☹ | ☺ | ☺ | ☺ | ☹ | ☺ | ☺ |
| Coates_2014 [33] | ? | ☺ | ☺ | ☹ | ? | ☺ | ☺ |
| Coates_2016 [34] | ? | ☺ | ☺ | ☹ | ? | ? | ☺ |
| Coates_2018 [35] | ☺ | ☺ | ☹ | ☺ | ? | ? | ? |
| Costa_2018 [36] | ☹ | ☺ | ☹ | ☺ | ? | ☺ | ? |
| Cretu_2018 [37] | ☺ | ☺ | ☺ | ☺ | ☺ | ☺ | ☺ |
| Dominguez_2009 [38] | ☹ | ? | ☺ | ☺ | ? | ? | ? |
| Duruoz_2018 [39] | ☺ | ☺ | ☺ | ? | ? | ☺ | ☺ |
| Fernández_Ávila_2017 [40] | ☹ | ☺ | ☺ | ☺ | ? | ☺ | ☺ |
| Ferreyra Garrott_2013 [41] | ? | ☺ | ☺ | ☺ | ? | ☺ | ☺ |
| Garg_2015 [42] | ☹ | ☺ | ? | ? | ☹ | ☺ | ? |
| Gladman_2009 [43] | ? | ☺ | ☺ | ☺ | ? | ☺ | ☺ |
| Haddad_2019 [44] | ? | ☺ | ☺ | ☺ | ? | ☺ | ☺ |
| Härle_2017 [45] | ☹ | ☺ | ☺ | ☹ | ☹ | ? | ? |
| Haroom_2013 [46] | ? | ☺ | ☺ | ☺ | ☹ | ? | ☺ |
| Husni_2007 [47] | ? | ☺ | ☺ | ? | ? | ☺ | ☺ |
| Husni_2014 [48] | ☹ | ☺ | ? | ? | ☹ | ☺ | ? |
| Ibrahim_2009 [49] | ? | ☺ | ? | ? | ? | ☺ | ? |
| Karreman_2017 [50] | ☹ | ☺ | ☺ | ☺ | ☹ | ☺ | ☺ |
| Khraishi_2010 [51] | ☹ | ☺ | ? | ☺ | ☺ | ☺ | ? |
| Khraishi_2011 [52] | ☺ | ☺ | ☹ | ☺ | ? | ☺ | ☹ |
| Leijten_2018 [53] | ☹ | ☺ | ☺ | ☺ | ? | ☺ | ☺ |
| López-Estebaranz_2015 [54] | ☺ | ☺ | ☺ | ☺ | ? | ☺ | ☺ |
| Maejima_2016 [55] | ? | ☺ | ☺ | ? | ? | ☺ | ☺ |
| Martire_2019 [56] | ☺ | ☺ | ☺ | ? | ☺ | ☺ | ☺ |
| Mazzotti_2019 [57] | ☺ | ☺ | ☺ | ☺ | ? | ☺ | ☺ |
| Mease_2014 [58] | ☺ | ☺ | ☹ | ☹ | ☺ | ? | ☹ |
| Mishra_2017 [59] | ☹ | ☺ | ☺ | ☺ | ☹ | ☺ | ☺ |
| Oyur_2014 [60] | ☹ | ☹ | ☺ | ☺ | ☹ | ☹ | ☺ |
| Piaserico_2016 [61] | ? | ☺ | ☺ | ? | ? | ☺ | ☺ |
| Salaffi_2018 [62] | ☹ | ☺ | ? | ☺ | ☹ | ☺ | ☺ |
| Tinazzi_2012 [63] | ☹ | ☺ | ☺ | ☺ | ☹ | ☺ | ☺ |
| Tom_2015 [64] | ☹ | ☺ | ☺ | ☹ | ☹ | ☺ | ☺ |
| Urbancek_2016 [65] | ☺ | ☺ | ☺ | ☹ | ☺ | ☺ | ☺ |
| Vidal_2016 [66] | ? | ☺ | ☹ | ? | ? | ☺ | ☹ |
| Walsh_2013 [67] | ☹ | ☺ | ☺ | ? | ☹ | ☺ | ☺ |
| You_2015 [68] | ☹ | ? | ☺ | ? | ☹ | ? | ☺ |

☺Low Risk ☹High Risk ? Unclear Risk

1. Bachelez H, Claudepierre P, Boulinguez S (2011) Psoriatic arthritis: A multidisciplinary approach. Early detection of psoriatic arthritis: What tools in practice? Annales de Dermatologie et de Venereologie 138 (6 SUPPL. 1):6-9. doi:10.1016/S0151-9638(11)70061-8

2. Barbour JA, Binding J, Bridges M, Kelly C (2003) Evaluation of a screening tool for inflammatory joint disease. Ann Rheum Dis 62 (2):187-188. doi:10.1136/ard.62.2.187

3. Biondi G, Addis G, Carcassi M, Taras L, Satta R, Montesu MA (2018) Early psoriatic arthritis in psoriatic patients: risk factors and screening for rheumatological evaluation. International Journal of Dermatology 57 (10):e97-e98. doi:10.1111/ijd.14136

4. Brzezińska O, Łuczak A, Małecki D, Kobiałka D, Lewandowska-Polak A, Poznańska-Kurowska K, Makowska J (2018) Assessment of musculoskeletal symptoms in patients with psoriasis. Alergia Astma Immunologia 23 (1):46-53

5. Busquets-Perez N, Marzo-Ortega H, McGonagle D, Waxman R, Helliwell P (2015) Screening psoriatic arthritis tools: analysis of the Early Arthritis for Psoriatic Patients questionnaire. Rheumatology (Oxford) 54 (1):200-202. doi:10.1093/rheumatology/keu426

6. Cantini F, Niccoli L, Nannini C, Kaloudi O, Bertoni M, Cassara E (2010) Psoriatic arthritis: a systematic review. International journal of rheumatic diseases 13 (4):300-317. doi:10.1111/j.1756-185X.2010.01540.x

7. Chandran V, Schentag CT, Gladman DD (2008) Sensitivity and specificity of the CASPAR criteria for psoriatic arthritis in a family medicine clinic setting. J Rheumatol 35 (10):2069-2070; author reply 2070

8. Choe YB, Park CJ, Yu DY, Kim Y, Ju HJ, Youn SW, Lee JH, Kim BS, Seo SJ, Yun SK, Park J, Kim NI, Youn JI, Lee SJ, Lee MG, Kim KJ, Ro YS, Song HJ, Shin BS, Ahn SK, Lee JY, Won YH, Jang MS, Kim KH, Kim MH, Kim TY, Choi JH (2019) Usefulness of the psoriatic arthritis screening and evaluation questionnaire to monitor disease activity in management of patients with psoriasis: Findings from the EPI-PSODE study. Annals of Dermatology 31 (1):29-36. doi:10.5021/ad.2019.31.1.29

9. Coates LC (2017) Patient education and screening for psoriatic arthritis is key in the care of patients with psoriasis, whichever method is chosen. British Journal of Dermatology 176 (3):574-575. doi:10.1111/bjd.15333

10. García-Gavín J, Pérez-Pérez L, Tinazzi I, Vidal D, Mc Gonagle D (2017) Spanish Cultural Adaptation of the Questionnaire Early Arthritis for Psoriatic Patients. Actas Dermo-Sifiliograficas 108 (10):924-930. doi:10.1016/j.adengl.2017.10.004

11. Gladman DD (2008) Can we identify psoriatic arthritis early? Current Rheumatology Reports 10 (6):419-421. doi:10.1007/s11926-008-0067-9

12. Henes JC, Ziupa E, Eisfelder M, Adamczyk A, Knaudt B, Jacobs F, Lux J, Schanz S, Fierlbeck G, Spira D, Horger M, Kanz L, Koetter I (2014) High prevalence of psoriatic arthritis in dermatological patients with psoriasis: a cross-sectional study. Rheumatol Int 34 (2):227-234. doi:10.1007/s00296-013-2876-z

13. Iragorri N, Hazlewood G, Manns B, Danthurebandara V, Spackman E (2019) Psoriatic arthritis screening: A systematic review and meta-analysis. Rheumatology (United Kingdom) 58 (4):692-707. doi:10.1093/rheumatology/key314

14. Jo SJ, Foley P, Oakley SP, Zhang J, Zheng M, Shin K, McGonagle D, Gisondi P, Tinazzi I, Butcher BE, Handel M (2019) Initial assessment of the early arthritis for psoriatic patients diagnostic questionnaire in dermatology clinics in Australia, Korea and China. International journal of rheumatic diseases 22 (8):1512-1520. doi:10.1111/1756-185X.13604

15. Khraishi M, Chouela E, Bejar M, Landells I, Hewhook T, Rampakakis E, Sampalis JS, Abouchehade K (2012) High prevalence of psoriatic arthritis in a cohort of patients with psoriasis seen in a dermatology practice. Journal of Cutaneous Medicine and Surgery 16 (2):122-127. doi:10.2310/7750.2011.10101

16. Mease PJ, Palmer JB, Hur P, Strober BE, Lebwohl M, Karki C, Reed GW, Etzel CJ, Greenberg JD, Helliwell PS (2019) Utilization of the validated Psoriasis Epidemiology Screening Tool to identify signs and symptoms of psoriatic arthritis among those with psoriasis: a cross-sectional analysis from the US-based Corrona Psoriasis Registry. J Eur Acad Dermatol Venereol 33 (5):886-892. doi:10.1111/jdv.15443

17. Merola JF, Husni ME, Qureshi AA (2013) Screening instruments for psoriatic arthritis. Journal of Rheumatology 40 (9):1623. doi:10.3899/jrheum.130474

18. Merola JF, Husni ME, Qureshi AA (2014) Psoriatic arthritis screening tools: Study design and methodological challenges. British Journal of Dermatology 170 (4):994-995. doi:10.1111/bjd.12815

19. Olivieri I, D'Angelo S, Padula A, Palazzi C (2008) The challenge of early diagnosis of psoriatic arthritis. Journal of Rheumatology 35 (1):3-5

20. Ravi D, Thomas J (2019) Significance of pase score in psoriasis: A cross-sectional study in a tertiary care hospital. Indian Journal of Public Health Research and Development 10 (8):1507-1508. doi:10.5958/0976-5506.2019.02303.9

21. Salaffi F, Di Carlo M, Bugatti L, Lato V, Nicolini M, Carotti M (2017) Development and pilot-testing of a new tool to screen psoriasis patients for the presence of psoriatic arthritis: the Simple Psoriatic Arthritis Screening (SiPAS) questionnaire. Journal of the European Academy of Dermatology and Venereology 31 (3):e167-e169. doi:10.1111/jdv.13902

22. Shin D, Kim HJ, Kim DS, Kim SM, Park JS, Park YB, Lee MG (2016) Clinical features of psoriatic arthritis in Korean patients with psoriasis: a cross-sectional observational study of 196 patients with psoriasis using psoriatic arthritis screening questionnaires. Rheumatol Int 36 (2):207-212. doi:10.1007/s00296-015-3365-3

23. Spelman L, Su JC, Fernandez-Peñas P, Varigos GA, Cooper AJ, Baker CS, Lee M, Ring JM, Thirunavukkarasu K (2015) Frequency of undiagnosed psoriatic arthritis among psoriasis patients in Australian dermatology practice. Journal of the European Academy of Dermatology and Venereology 29 (11):2184-2191. doi:10.1111/jdv.13210

24. Tay SH, Lim AY, Lee TL, Low BP, Cheung PP (2014) The value of referral letter information in predicting inflammatory arthritis--factors important for effective triaging. Clin Rheumatol 33 (3):409-413. doi:10.1007/s10067-014-2503-8

25. Taylor SL, Petrie M, O'Rourke KS, Feldman SR (2009) Rheumatologists' recommendations on what to do in the dermatology office to evaluate and manage psoriasis patients' joint symptoms. Journal of Dermatological Treatment 20 (6):350-353. doi:10.3109/09546630902817887

26. Wernham AGH, Jobanputra P (2015) Dermatologists seeking rheumatologists: An analysis of referrals of patients with psoriasis to a rheumatology service. Rheumatology (United Kingdom) 54 (10):1929-1930. doi:10.1093/rheumatology/kev230

27. Wittkowski KM, Leonardi C, Gottlieb A, Menter A, Krueger GG, Tebbey PW, Belasco J, Soltani-Arabshahi R, Gray J, Horn L, Krueger JG, The International Psoriasis C (2011) Clinical symptoms of skin, nails, and joints manifest independently in patients with concomitant psoriasis and psoriatic arthritis. PLoS ONE 6 (6). doi:10.1371/journal.pone.0020279

28. Audureau E, Roux F, Lons Danic D, Bagot M, Cantagrel A, Dernis E, Gouyette N, Hilliquin P, Jullien D, Liote F, Passeron T, M AR, Claudepierre P (2018) Psoriatic arthritis screening by the dermatologist: development and first validation of the 'PURE-4 scale'. J Eur Acad Dermatol Venereol 32 (11):1950-1953. doi:10.1111/jdv.14861

29. Chandran V, Gladman DD (2011) Toronto Psoriatic Arthritis Screening (ToPAS) questionnaire: A report from the GRAPPA 2009 Annual Meeting. Journal of Rheumatology 38 (3):546-547. doi:10.3899/jrheum.101117

30. Chimenti MS, Esposito M, Graceffa D, Teoli M, Peluso G, Birra D, Moretta G, Galossi A, Carboni V, Sensi F, Mazzotta A, Caccavale R, Bernardini N, Sessa P, Richetta A, Del Duca E, Urbani S, Persechino S, De Simone C, Bonifati C, Gremese E, Peris K, Perricone R (2019) PsA-Disk, a novel visual instrument to evaluate psoriatic arthritis in psoriatic patients: an Italian derma-rheuma multicentre study. Ther Adv Chronic Dis 10:2040622319847056. doi:10.1177/2040622319847056

31. Chiowchanwisawakit P, Wattanamongkolsil L, Srinonprasert V, Petcharat C, Siriwanarangsun P, Katchamart W (2016) Developing the Thai Siriraj Psoriatic Arthritis Screening Tool and validating the Thai Psoriasis Epidemiology Screening Tool and the Early Arthritis for Psoriatic Patients questionnaire. Rheumatol Int 36 (10):1459-1468. doi:10.1007/s00296-016-3513-4

32. Coates LC, Aslam T, Al Balushi F, Burden AD, Burden-Teh E, Caperon AR, Cerio R, Chattopadhyay C, Chinoy H, Goodfield MJ, Kay L, Kelly S, Kirkham BW, Lovell CR, Marzo-Ortega H, McHugh N, Murphy R, Reynolds NJ, Smith CH, Stewart EJ, Warren RB, Waxman R, Wilson HE, Helliwell PS (2013) Comparison of three screening tools to detect psoriatic arthritis in patients with psoriasis (CONTEST study). The British journal of dermatology 168 (4):802-807. doi:10.1111/bjd.12190

33. Coates LC, Walsh J, Haroon M, FitzGerald O, Aslam T, Al Balushi F, Burden AD, Burden-Teh E, Caperon AR, Cerio R, Chattopadhyay C, Chinoy H, Goodfield MJ, Kay L, Kelly S, Kirkham BW, Lovell CR, Marzo-Ortega H, McHugh N, Murphy R, Reynolds NJ, Smith CH, Stewart EJ, Warren RB, Waxman R, Wilson HE, Helliwell PS (2014) Development and testing of new candidate psoriatic arthritis screening questionnaires combining optimal questions from existing tools. Arthritis Care Res (Hoboken) 66 (9):1410-1416. doi:10.1002/acr.22284

34. Coates LC, Savage L, Waxman R, Moverley AR, Worthington S, Helliwell PS (2016) Comparison of screening questionnaires to identify psoriatic arthritis in a primary-care population: a cross-sectional study. The British journal of dermatology 175 (3):542-548. doi:10.1111/bjd.14604

35. Coates LC, Savage LJ, Chinoy H, Laws PM, Lovell CR, Korendowych E, Mahmood F, Mathieson HR, McGonagle D, Warren RB, Waxman R, Helliwell PS (2018) Assessment of two screening tools to identify psoriatic arthritis in patients with psoriasis. J Eur Acad Dermatol Venereol 32 (9):1530-1534. doi:10.1111/jdv.14971

36. Costa CZ, Goldenstein-Schainberg C, Carneiro S, Rodrigues JJ, Romiti R, Barros TBM, Martins G, Carneiro J, Grynszpan R, Sampaio AL, Mendonca TMS, Silva CHM, Qureshi AA, Pinto RMC, Ranza R (2018) Semantic and psychometric validation of the Brazilian Portuguese version (PASE-P) of the Psoriatic Arthritis Screening and Evaluation questionnaire. PLoS One 13 (10):e0205486. doi:10.1371/journal.pone.0205486

37. Cretu D, Gao L, Liang K, Soosaipillai A, Diamandis EP, Chandran V (2018) Differentiating Psoriatic Arthritis From Psoriasis Without Psoriatic Arthritis Using Novel Serum Biomarkers. Arthritis Care Res (Hoboken) 70 (3):454-461. doi:10.1002/acr.23298

38. Dominguez PL, Husni ME, Holt EW, Tyler S, Qureshi AA (2009) Validity, reliability, and sensitivity-to-change properties of the psoriatic arthritis screening and evaluation questionnaire. Archives of dermatological research 301 (8):573-579. doi:10.1007/s00403-009-0981-3

39. Duruoz MT, Sanal Toprak C, Ulutatar F (2018) Validation of the Toronto Psoriatic Arthritis Screen II (TOPAS II) questionnaire in a Turkish population. Rheumatol Int 38 (2):255-259. doi:10.1007/s00296-017-3871-6

40. Fernández-Ávila DG, Beltrán A, González C, Castro L, Rincón-Riaño DN, Díaz MC, Gutiérrez JM (2017) Translation and validation of the Spanish version of the ToPAS (Toronto Psoriatic Arthritis Screening) questionnaire for use on patients with psoriatic arthritis in Dermatology clinics in Colombia. Revista Colombiana de Reumatologia 24 (2):79-83. doi:10.1016/j.rcreu.2016.12.005

41. Ferreyra Garrott LG, Soriano ER, Rosa JE, Navarta DA, Saucedo C, Scolnik M, Bedran Z, Sabelli M, Garcia MV, Anselmi C, Galimberti R, Catoggio LJ, Husni ME, Qureshi AA (2013) Validation in Spanish of a screening questionnaire for the detection of psoriatic arthritis in patients with psoriasis. Rheumatology (Oxford) 52 (3):510-514. doi:10.1093/rheumatology/kes306

42. Garg N, Truong B, Ku JH, Devere TS, Ehst BD, Blauvelt A, Deodhar AA (2015) A novel, short, and simple screening questionnaire can suggest presence of psoriatic arthritis in psoriasis patients in a dermatology clinic. Clin Rheumatol 34 (10):1745-1751. doi:10.1007/s10067-014-2658-3

43. Gladman DD, Schentag CT, Tom BD, Chandran V, Brockbank J, Rosen C, Farewell VT (2009) Development and initial validation of a screening questionnaire for psoriatic arthritis: the Toronto Psoriatic Arthritis Screen (ToPAS). Ann Rheum Dis 68 (4):497-501. doi:10.1136/ard.2008.089441

44. Haddad A, Feld J, Zisman D (2019) The Performance of Psoriatic Arthritis Screening Questionnaires in Patients with Psoriasis. J Rheumatol 46 (12):1643-1645. doi:10.3899/jrheum.180939

45. Härle P, Letschert K, Wittig B, Mrowietz U (2016) Sensitivity of the GEPARD Patient Questionnaire to Identify Psoriatic Arthritis in Patients with Psoriasis in Daily Practice: The GEPARD-Life Study. Dermatology 232 (5):597-605. doi:10.1159/000448029

46. Haroon M, Kirby B, FitzGerald O (2013) High prevalence of psoriatic arthritis in patients with severe psoriasis with suboptimal performance of screening questionnaires. Ann Rheum Dis 72 (5):736-740. doi:10.1136/annrheumdis-2012-201706

47. Husni ME, Meyer KH, Cohen DS, Mody E, Qureshi AA (2007) The PASE questionnaire: Pilot-testing a Psoriatic Arthritis Screening and Evaluation tool. Journal of the American Academy of Dermatology 57 (4):581-587. doi:10.1016/j.jaad.2007.04.001

48. Husni ME, Qureshi AA, Koenig AS, Pedersen R, Robertson D (2014) Utility of the PASE questionnaire, psoriatic arthritis (PsA) prevalence and PsA improvement with anti-TNF therapy: results from the PRISTINE trial. The Journal of dermatological treatment 25 (1):90-95. doi:10.3109/09546634.2013.800185

49. Ibrahim GH, Buch MH, Lawson C, Waxman R, Helliwell PS (2009) Evaluation of an existing screening tool for psoriatic arthritis in people with psoriasis and the development of a new instrument: the Psoriasis Epidemiology Screening Tool (PEST) questionnaire. Clinical and experimental rheumatology 27 (3):469-474

50. Karreman MC, Weel A, van der Ven M, Vis M, Tchetverikov I, Nijsten TEC, Wakkee M, Hazes JMW, Luime JJ (2017) Performance of screening tools for psoriatic arthritis: a cross-sectional study in primary care. Rheumatology (Oxford) 56 (4):597-602. doi:10.1093/rheumatology/kew410

51. Khraishi M, Landells I, Mugford G (2010) The Self-Administered Psoriasis and Arthritis Screening Questionnaire (PASQ): A Sensitive and Specific Tool for the Diagnosis of Early and Established Psoriatic Arthritis. Psoriasis forum 16 (2):9-16

52. Khraishi M, Mong J, Mugford G, Landells I (2011) The electronic Psoriasis and Arthritis Screening Questionnaire (ePASQ): a sensitive and specific tool to diagnose psoriatic arthritis patients. J Cutan Med Surg 15 (3):143-149. doi:10.2310/7750.2011.10018

53. Leijten EFA, Sigurdsson V, Wenink MH, Radstake TRDJ (2017) Screening for psoriatic arthritis using the Psoriasis Epidemiology Screening Tool questionnaire: examining the optimal cut-off. British Journal of Dermatology 176 (5):1357-1359. doi:10.1111/bjd.14953

54. Lopez Estebaranz JL, Zarco-Montejo P, Samaniego ML, Garcia-Calvo C (2015) Prevalence and clinical features of psoriatic arthritis in psoriasis patients in Spain. Limitations of PASE as a screening tool. European journal of dermatology : EJD 25 (1):57-63. doi:10.1684/ejd.2014.2449

55. Maejima H, Katayama C, Taniguchi T, Aki R, Nishiyama H, Yanagita K, Sato Y, Tinazzi I, Watarai A, Amoh Y (2016) Japanese version of the early psoriatic arthritis screening questionnaire (EARP). Journal of Dermatology 43 (4):385-388. doi:10.1111/1346-8138.13092

56. Martire MV, Girard Bosch MP, Scarafia S, Cosentino V, Tapia Moreira MJ, Estrella N, Marín J, Sommerfleck F, Maldonado Ficco H, Catay ER, Benegas M, Kerzberg E, Soriano ER (2019) Spanish Validation of the GEPARD Questionnaire for the Detection of Psoriatic Arthritis in Argentinian Patients with Psoriasis. Dermatology 235 (2):101-106. doi:10.1159/000495983

57. Mazzotti NG, Palominos PE, Bredemeier M, Kohem CL, Cestari TF (2019) Cross-cultural validation and psychometric properties of the Brazilian Portuguese version of the Psoriasis Epidemiology Screening Tool (PEST-bp). Archives of dermatological research. doi:10.1007/s00403-019-02013-9

58. Mease PJ, Gladman DD, Helliwell P, Khraishi MM, Fuiman J, Bananis E, Alvarez D (2014) Comparative performance of psoriatic arthritis screening tools in patients with psoriasis in European/North American dermatology clinics. J Am Acad Dermatol 71 (4):649-655. doi:10.1016/j.jaad.2014.05.010

59. Mishra S, Kancharla H, Dogra S, Sharma A (2017) Comparison of four validated psoriatic arthritis screening tools in diagnosing psoriatic arthritis in patients with psoriasis (COMPAQ Study). The British journal of dermatology 176 (3):765-770. doi:10.1111/bjd.14929

60. Oyur KB, Engin B, Hatemi G, Asma A, Kutlubay Z, Bulut N, Serdaroglu S, Tuzun Y (2014) Turkish PASE: Turkish Version of the Psoriatic Arthritis Screening and Evaluation Questionnaire. Ann Dermatol 26 (4):457-461. doi:10.5021/ad.2014.26.4.457

61. Piaserico S, Gisondi P, Amerio P, Amoruso G, Campanati A, Conti A, De Simone C, Gualdi G, Guarneri C, Mazzotta A, Musumeci ML, Abeni D (2016) Validation and field performance of the Italian version of the psoriatic arthritis screening and evaluation (PASE) questionnaire. Acta Dermato-Venereologica 96:96-101. doi:10.2340/00015555-2429

62. Salaffi F, Di Carlo M, Luchetti MM, Di Donato E, Campanati A, Benfaremo D, Nicolini M, Carotti M, Giacchetti A, Ganzetti G, Bugatti L, Gabrielli A, Offidani AM (2018) A validation study of the Simple Psoriatic Arthritis Screening (SiPAS) questionnaire to screen psoriasis patients for psoriatic arthritis. Clinical and experimental rheumatology 36 (1):127-135

63. Tinazzi I, Adami S, Zanolin EM, Caimmi C, Confente S, Girolomoni G, Gisondi P, Biasi D, McGonagle D (2012) The early psoriatic arthritis screening questionnaire: a simple and fast method for the identification of arthritis in patients with psoriasis. Rheumatology (Oxford) 51 (11):2058-2063. doi:10.1093/rheumatology/kes187

64. Tom BD, Chandran V, Farewell VT, Rosen CF, Gladman DD (2015) Validation of the Toronto Psoriatic Arthritis Screen Version 2 (ToPAS 2). J Rheumatol 42 (5):841-846. doi:10.3899/jrheum.140857

65. Urbancek S, Sutka R, Kmecova Z, Salkovska J, Vano I, Pecova T, Rovensky J (2016) Screening of Patients with Psoriasis for Psoriatic Arthritis in the Slovak Republic. Acta Medica Martiniana 16 (3):32-42. doi:10.1515/acm-2016-0015

66. Vidal D, Reina D, Martin JL, Cerda D, Estrada P, Garcia-Diaz S, Navarro V, Peramiquel L, Roig D, Torrente V, Corominas H (2016) PASE and EARP questionnaires for the identification of enthesitis, synovitis, and tenosynovitis in patients with psoriasis. Clin Rheumatol 35 (10):2463-2468. doi:10.1007/s10067-016-3392-9

67. Walsh JA, Callis Duffin K, Krueger GG, Clegg DO (2013) Limitations in screening instruments for psoriatic arthritis: a comparison of instruments in patients with psoriasis. J Rheumatol 40 (3):287-293. doi:10.3899/jrheum.120836

68. You HS, Kim GW, Cho HH, Kim WJ, Mun JH, Song M, Kim HS, Ko HC, Kim MB, Lee SG, Lee IS, Kim BS (2015) Screening for Psoriatic Arthritis in Korean Psoriasis Patients Using the Psoriatic Arthritis Screening Evaluation Questionnaire. Ann Dermatol 27 (3):265-268. doi:10.5021/ad.2015.27.3.265
