# Supplementary material for: Psoriatic arthritis screening: A systematic literature review and experts’ recommendations
Source: PLoS One. 2021 Mar 15;16(3):e0248571. doi: 10.1371/journal.pone.0248571 (PMC7959352; doi:10.1371/journal.pone.0248571)
Supplement: Attachment — Submitted filename: Response to Reviewers.docx [file pone.0248571.s003.docx]

Manuscript [PONE-D-20-25482] - [EMID:631d5c122f91803b]

Barcelona, December 18^th^, 2020

Dear editor,

Thank you very much for the opportunity to revise our manuscript by answering the reviewers´comments. And thank you as well to the reviewers for the improving the readiness of our manuscript with their suggestions. We have replied to each one of them, as you can see below.

We hope, these responses and changes clarify the study.

Sincerely,

Jordi Gratacós on behalf of the authors.

Reviewer #1: The paper is interesting, well written and there is no such paper in the literature, it fit with purpose of the journal and seems of interest for the readers.

I only suggest professional revision by a statistician.

Thank you for your comment. We have consulted a expert statistician and we have made some changes.

Reviewer #2: Dear authors, you report in an exhausting way the relationship between the implementation of PsA screening tools and PSA prognosis. Moreover, study design and table are easy to understand, clear and adequate. I report some notes to improve your manuscript:

Thank you very much

1) Implement introduction and relation between pso and psa from:
Caputo V, Strafella C, Termine A, Dattola A, Mazzilli S, Lanna C, Cosio T, Campione E, Novelli G, Giardina E, Cascella R. Overview of the molecular determinants contributing to the expression of Psoriasis and Psoriatic Arthritis phenotypes. J Cell Mol Med. 2020 Oct 31. doi: 10.1111/jcmm.15742. Epub ahead of print. PMID: 33128843.

Thank you very much for your recommendation.

2) Line 151, 298, of, correct

Done.

3) Page 20, correct caption

Done

4) Page 25, line 281-290. Did you consider national center for psoriasis? General hospitals’ attended populations could not reflect PSA center population.

Thank you for your comment. We have made some changes.

*Most were teaching hospitals (about half of them are considered referred centers) of different sizes (14% were county hospitals)*

5) Table 5, this is a hotspot, really interesting.

Thank you for your comment

6) Pag 27 line 323-327. Other consideration in timing?

We have added some information
